# Metabolomic Characterizations of Liver Injury Caused by Acute Arsenic Toxicity in Zebrafish

**DOI:** 10.1371/journal.pone.0151225

**Published:** 2016-03-11

**Authors:** Caixia Li, Ping Li, Yee Min Tan, Siew Hong Lam, Eric C. Y. Chan, Zhiyuan Gong

**Affiliations:** 1 Department of Biological Sciences, National University of Singapore, Singapore, Singapore; 2 NUS Environmental Research Institute, National University of Singapore, Singapore, Singapore; 3 Department of Pharmacy, National University of Singapore, Singapore, Singapore; Chinese Academy of Sciences, CHINA

## Abstract

Arsenic is one of the most common metalloid contaminants in groundwater and it has both acute and chronic toxicity affecting multiple organs. Details of the mechanism of arsenic toxicity are still lacking and profile studies at metabolic level are very limited. Using gas chromatography coupled with mass spectroscopy (GC/MS), we first generated metabolomic profiles from the livers of arsenic-treated zebrafish and identified 34 significantly altered metabolite peaks as potential markers, including four prominent ones: cholic acid, glycylglycine, glycine and hypotaurine. Combined results from GC/MS, histological examination and pathway analyses suggested a series of alterations, including apoptosis, glycogenolysis, changes in amino acid metabolism and fatty acid composition, accumulation of bile acids and fats, and disturbance in glycolysis related energy metabolism. The alterations in glycolysis partially resemble Warburg effect commonly observed in many cancer cells. However, cellular damages were not reflected in two conventional liver function tests performed, Bilirubin assay and alanine aminotransferase (ALT) assay, probably because the short arsenate exposure was insufficient to induce detectable damage. This study demonstrated that metabolic changes could reflect mild liver impairments induced by arsenic exposure, which underscored their potential in reporting early liver injury.

## Introduction

Arsenic is one of the most common metalloid toxicants contaminating groundwater and this contamination is a global concern. Two inorganic forms of arsenic, trivalent (As^3+^) and pentavalent (As^5+^), are the main forms in groundwater, with As^5+^ as the predominant form in oxidizing conditions [1]. Inorganic arsenic is both acutely and chronically toxic and it is classified as a carcinogen by the International Agency for Research on Cancer [2]. Long-term exposure to arsenic is associated with increased risk of cancers in liver, skin, lungs, bladder and kidney. It can also disrupt the cardiovascular, reproduction, nervous system and immune system [3–5]. 10 μg/L has been set by World Health Organization as the maximal level for arsenic contaminant in drinking water, but millions of people worldwide, particularly in some Asian countries, are still exposed to toxic levels (>50 μg/L) due to surface water and groundwater contamination [1]. Molecular mechanisms of arsenic toxicity have been long studied. The involvement of oxidative stress, genotoxicity, altered DNA methylation and others have been reported; however, details of the mechanism of action through an integrated approach are still lacking [6].

Applications of omic approaches in aquatic toxicology have been rapidly increasing as a result of technological development and availability of genome sequences for common aquatic model organisms (e.g. zebrafish, medaka, fathead minnow, and water flea). For arsenic exposure in human, there have been many publications on transcriptome but only a few studies have been conducted on proteome [7] and one on metabolome (mixed exposure to arsenic, cadmium and lead) [8]. Similarly in non-human studies, metabolomic studies on arsenic toxicity are scarce; so far only four reports are on rodents [9–12] and two reports on non-rodents, clam [13] and Daphnia [14]. Despite the importance of fish models in aquatic toxicology, arsenic-induced metabolomic changes in fish have not yet been reported.

Among the fish models, the zebrafish (*Danio rerio*) has a unique combination of advantageous traits for scientific research. It has evolutionarily conserved genes and molecular pathways when compared to higher vertebrates, well developed genetic manipulation tools and a large body of literature data, including the complete genome sequences to facilitate genome-wide studies [15]. Studies of arsenic using the zebrafish embryos have reported substantial toxicity during embryogenesis [16], neural defects [17], vascular development [18], immune response [19, 20]. Fewer studies have been performed on adult zebrafish, which usually reported molecular events such as cellular transport of arsenic [21], transcriptomic changes and biomarker gene selection [22], and changes of molecules or pathways of interest [23]. Previously, we have used adult zebrafish to investigate arsenic-induced transcriptomic changes in the liver, the primary detoxification site and target organ of arsenic [3, 24]. In this study, we aimed to assess the changes in liver metabolic profile after 96-hr acute exposure to inorganic arsenic (As^5+^) using gas chromatography coupled with mass spectroscopy (GC/MS). We identified 34 significantly altered metabolite peaks as potential marker. Liver damages implied by the metabolomic changes were further validated by histological examination but not reflected in two conventional liver function tests. Our study indicated that metabolic markers may be of great potential in reporting early liver damages.

## Material and Methods

### Acute arsenic exposure in adult zebrafish

The use of zebrafish in this study was in strict accordance with the recommendations in the Guide for the Care and Use of Laboratory Animals of the National Institutes of Health. The protocol was approved by the Institutional Animal Care and Use Committee (IACUC) of the National University of Singapore (Protocol Number: 096/12). Adult male zebrafish of Singapore wildtype strain, aged 6–10 months, were purchased from Mainland Tropical Fish Farm, Singapore and acclimatized for two weeks. Standard 96-hr acute exposures to sodium arsenate (Na_2_HAsO_4_•7H_2_O, Sigma) at 20 ppm (for metabolomic profiling) or at 15 ppm (for validation of robust changes at slightly lower concentration) were conducted in replicate tanks of 15 fish each. The control groups were similarly set up without arsenate. Media were renewed once on day 3 of exposure experiment. The water condition and health status of fish were monitored daily. Fish with signs of unrelieved sickness (e.g. inactive, reduced breathing, and staying at only bottom of tank or surface of water) were euthanized with 250 mg/L MS222. At the end of treatment, fish were euthanized in ice water instead of anesthesia to minimized potential undesirable changes induced on liver metabolome. For metabolic profiling, liver samples were collected as a pool of four livers per replicate and five replicates per group, snap-frozen in liquid nitrogen, and then stored at -80°C until metabolite extraction. Sampling for histological examination and liver function tests are described in separate sections.

### Metabolite sample preparation

Metabolite samples were prepared following an established protocol [25] with optimization to maximize extraction from liver. Briefly, 20 mg tissues were homogenized in water (1:10 w/v) using pulse sonication. After a brief centrifugation at 4°C, 100 μL of supernatant was transferred to 1 mL of extraction buffer, a solvent mixture of chloroform-methanol-water (2:5:2 v/v/v) with 0.5 mg/L diclofenac as internal standard, for metabolite extraction in a large capacity mixer for 30 min and centrifuged at 10,000 *g* at 4°C for 10 min. 800 μL supernatant was aspirated into a salinized glass tube and dried in TurboVap LV nitrogen evaporator (Caliper Life Science) at 50°C for 1 hr. To further dehydrate the extract, 100 μL of toluene (dried over Na_2_SO_4_) was added, vortex-mixed for 1 min and dried in TurboVap. Metabolites were reconstituted in 40 μl methoxyamine hydrochloride (MOX) for methoxymation at 60°C for 2 hr. For silylation, 60 μL N-Methyl-N-(trimethylsilyl) trifluoroacetamide (MSTFA; Alpha Analytical) was added and incubated at 70°C for 30 min. Processed samples were then transferred to GC vials for GC/MS analysis. For quality assessment of procedures, after pulse sonication in water, 80 μl aliquot from each control replicate was taken and well mixed, from which three 100-μL aliquots were used as quality control (QC) samples, which were then processed in exactly the same way as other samples throughout the experiment.

### GC/MS metabolic profiling

GC/MS was performed using a Pegasus 4D TOFMS (LecoCorp) equipped with an Agilent 7890 GC and a CTC CombiPAL autosampler. A DB-1 capillary column (30 m × 250 μm (i.d.) × 0.25 μm) with DuraGuard (Agilent Technologies J&W) was used. Helium flow rate was set at 1.5 mL/min and the injection volume was 1 μL with injector split ratio 1:20. For GC, the temperature was 220°C for front inlet and 280°C for transfer line. Column temperature was programed to be at 70°C for 0.2 min, ramped at 15°C/min to 270°C and then at 40°C/min to 310°C, and finally hold at 310°C for 8 min. For MS, the detector voltage was 1,600 V with an acquisition delay of 200 seconds. To facilitate metabolite identification, alkane standard mix (C10-C40; Sigma) and FAME (fatty acid methyl esters) standards (C8-C28; Sigma) were analyzed using the same settings, so that the GC retention time could be converted to two types of retention index (RI), Kovats index and Fiehn index, respectively.

### Data processing

The GC/MS spectra were processed with LECO’s ChromaTOF software for peak picking (S/N = 100, peakwidth modulation 2.5 s), tentative metabolite identification by MS spectra matching with library compounds (similarity score>600), peak alignment, and calculation of RIs. All de-convoluted chromatographs were manually inspected for consistent peak area integration. Missing values were filled by integration of baseline in chromatograph. Peak areas were normalized using quantile normalization (QN), where metabolites were ranked and individual values were substituted with reference to the same ranks in other samples following defined steps. It is expected to make distributions identical in statistical properties [26]. Normalized peaks were cleaned by discarding peaks with coefficient of variance (CV) >0.3 in QC replicates [25] before chemometric analysis.

### Chemometric analysis

Peaks (CV<0.3) were subjected to Principle Component Analysis (PCA), Partial Least Square-Discriminant Analysis (PLS-DA) modeling in SIMCA-P software (version 11, Umetrics) in addition to Welch’s *t-*test. PLS-DA model was validated by 1,000 rounds of built-in permutation test. Metabolites with variable importance plot values (VIPs) greater than 1.0 in PLS-DA model was considered to be positively contributing to the group separation. Peaks with VIP>1 and *p*<0.05 in *t*-test were considered as potential markers.

### Identification of metabolites and biological interpretation

Tentative identities of metabolites were obtained by spectra matching as above described. For peaks with VIP>1, spectra hits were inspected and identity of the corresponding underivatized structures were confirmed using ChemSpider (http://www.chemspider.com/). RIs were compared to those in the following databases: NIST Chemistry WebBook (http://webbook.nist.gov/chemistry/), Human Metabolite Database (HMDB, http://www.hmdb.ca/), Golm metabolome database (http://gmd.mpimp-golm.mpg.de/) and Metabolomics Workbench (http://www.metabolomicsworkbench.org/). Identification status of a metabolite was considered “confirmed” when: (1) its spectrum was highly similar to an item in a library; and (2) both RIs were consistent with literature values. Identification status was considered “probable” when (1) its spectrum was highly similar to an item in library; (2) only one RI was available in literature and the calculated RI was consistent with it. Other metabolites were denoted as “unknown”. Metabolites with “confirmed” and “probable” identities were input into Ingenuity Pathway Analysis (IPA) for biological interpretation.

### Histological examination

Fish were euthanized by ice and fixed in either 10% formalin (for paraffin section; Sigma) or 4% PFA (for cryosection; Sigma). For paraffin section, fixed samples were gradually dehydrated in ethanol, exchanged into Histoclear (National Dianogstics), and embedded into paraffin (Sigma). 5 μm sagittal sessions of livers were taken for Hematoxylin & Eosin (H&E) staining, Periodic acid–Schiff (PAS) staining and Tunnel assay. For cryosection, fixed samples were embedded in 1.5% bactoagar containing 5% sucrose and mounted to a cryostat microtome holder in tissue-freezing medium. 8 μm sections were used for Oil Red O (ORO) staining.

Parrafin sections were de-parrafinized in HistoClear and rehydrated to water before staining. For H&E staining, sections were stained in Mayer’s Hematoxylin (Sigma), gradually dehydrated to 90% alcohol, and counterstained in Eosin (Sigma). For PAS staining, sections were oxidized in 0.5% periodic acid (Sigma), incubated in Schiff’s reagent (Sigma), and washed in lukewarm water before counterstaining in Mayer’s hematoxylin. Tunnel assay was performed using In situ Cell Death Detection Kit, Fluorescein (Roche) according to manufacturer’s protocol. For ORO staining, cryosection slides were incubated in absolute propylene glycol (Sigma), stained in 0.5% ORO (Sigma) at 60°C, rinsed in 85% propylene glycol and then in water, and finally briefly counterstained in Mayer’s Hematoxylin.

### Liver function tests

Bilirubin assay kit and alanine aminotransferase (ALT) activity assay kit (Sigma) were used to assess functional status of fish liver. For bilirubin assay, only plasma was used. For ALT activity assay, both plasma and liver protein extracts were used.

Whole blood was collected at the dorsal aorta after tail ablation using P20 micropipettor fitted to an elongated tip (Prot/Elec Tips, Bio-Rad) and aspired into pre-chilled Eppendorf tubes. Both pipette tips and tubes were pre-coated with EDTA (submerging in 18 mg/mL EDTA solution for one day and then dried). Blood of 3–6 fish were pooled as one sample. Plasma was obtained as clear supernatant after centrifugation at 1000 *g* for 10 min at 4°C and stored at -80°C. Bilirubin assay was completed within 2 days of plasma collection.

To extract total protein, liver tissue was homogenized in ALT assay buffer (1:15 w/v) on ice and then centrifuged at 14,000 rpm at 4°C for 10 min. Supernatant was collected. Protein concentration was estimated based on Bradford method using Bio-Rad Protein Assay Dye Reagent and Bovine Serum Albumin standard (Sigma). ALT assay was performed following manufacturer’s protocol, while bilirubin assay was scaled down after confirming the linearity of bilirubin standard (S1 Fig).

## Results and Discussion

Both As^3+^ and As^5+^ are present in environmental water and of concern [1]. In this study, we selected As^5+^ for metabolomic studies because of availability of transcriptomic data we previously generated on As^5+^ exposed zebrafish livers [3, 24] for integrated studies. Fish exposed to 20 ppm and 15 ppm were more lethargic than those in control groups. Survival by the end of exposure was ~80% (25/30) at 20 ppm and 100% (30/30) at 15 ppm. Arsenic is known to be easily absorbed in gastrointestinal tract and biotransformed in liver before distribution to various other organs [27]. The liver is the main organ for bioaccumulation of arsenic in fish and the bioconcentration factors are between 3–13 fold [21, 28–30]. Metabolic profiles of livers from male adult zebrafish in both control and 20 ppm arsenic (As^5+^)-treated group were obtained using GC/MS, in which 331 metabolite peaks were confidently detected. The peak areas (representing abundance of the metabolites) were quantile-normalized and 239 reliable peaks (CV<0.3 within QC samples) were used for chemometric analysis.

### Arsenate treatment changed liver metabolic profile

**Fig 1:**
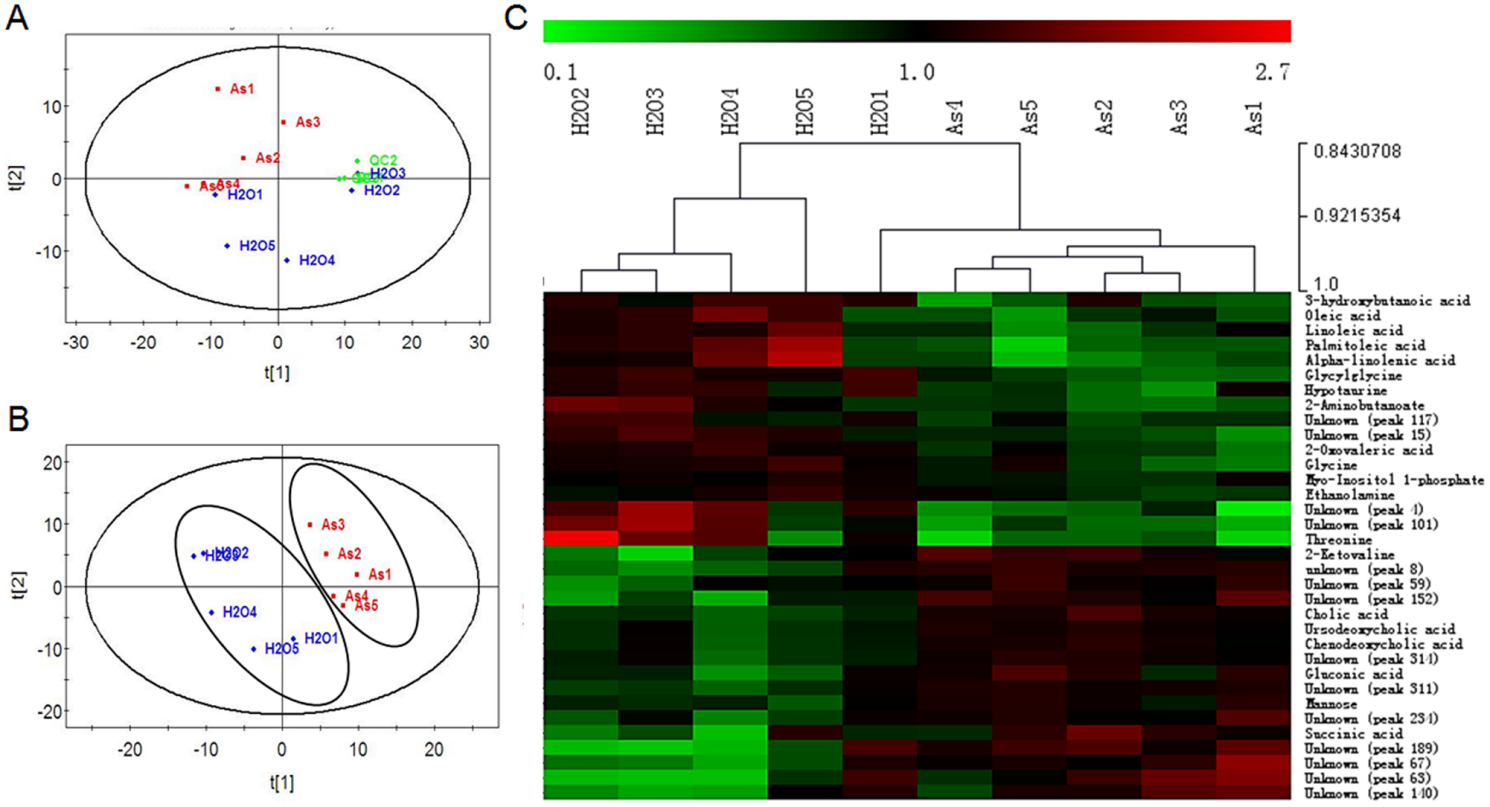
Results of chemometric analysis and hierarchical clustering. (A) PCA plot showing tight clustering of QC samples and inherent separation between control and treated groups. (B) PLS-DA plot with decent separation of the two groups. (C) Hierarchical clustering of samples using peak areas of the 34 potential metabolite markers. Peak areas were quantile-normalized and standardized to respective mean value of each metabolite among all samples. Hierarchical clustering was performed using Pearson correlation method without mean centering.

The 239 peaks were input to SIMCA-P software for PCA and PLS-DA modeling. In PCA plot, three QC samples were clustered tightly among themselves (and with two control replicates), suggesting good quality of sample processing and reproducibility of GC/MS (Fig 1A); however, the spread of replicates in both control and As groups indicated high biological variations. By PLS-DA modeling, a decent separation of the two groups, largely based on the first component, was observed (Fig 1B). There were 110 metabolites with VIP>1, 48 increased and 62 decreased in the As group. From these metabolites, 57 unique metabolites were identified, 25 of which were with status as “confirmed” and 32 as “probable” (S1 Table). It should be pointed out that, the distance between control and As groups in modeling was small, implying that exposure to 20 ppm As^5+^ caused significant but not extensive changes to the profiles.

To identify potential markers, Welch’s t-test was applied to the 110 peaks and 34 of them had p<0.05 (Table 1). Hierarchical clustering of these 34 potential markers (Fig 1C) revealed similar patterns among the As replicates. Patterns of control replicates were more variable and one replicate (H2O1) was closer to the As cluster than to the control cluster, which was consistent with the PCA plot. Yet, with a closer examination, the two groups could be distinguished even with only seven selected potential markers (S2 Fig). 20 out of the 34 potential markers were identified (confirmed or probable), among which cholic acid (FC = 1.51, p = 0.002) and glycylglycine (FC = 0.57, *p* = 0.0002) showed best distinction between the two groups in the clustering. Other notable markers were hypotaurine (decreased), four decreased unsaturated lipids (linoleic acid, alpha-linolenic acid, palmitoleic acid, and oleic acid), three decreased amino acids (threonine, glycine and 2-aminobutanoate), and two increased bile acids (ursodeoxycholic acid and chenodeoxycholic acid). Glycylglycine (diglycine) is the simplest dipeptide, whose source can be endogenous or exogenous. Hypotaurine is an intermediate in the synthesis of taurine from cysteine. In liver, bile acids need to be conjugated with taurine or glycine to form bile salts for secretion. The concurrent decrease of hypotaurine and glycine may contribute to the accumulation of bile acids. These effects of arsenic on bile acids and hypotaurine in the liver have not been reported before. For glycine, a recently study reported reduction of glycine in mouse liver by co-treatment of high fat diet (HFD) and sodium arsenite as compared to HFD alone, but no difference was observed between low fat diet groups [31]. Therefore, glycylglycine, glycine, hypotaurie and cholic acid may be promising candidate marker of arsenic toxicity. Furthermore, these four metabolites have been detected in urine and/or plasma of human and rat [32–34], making non-invasive assessment possible.

**Table 1:** List of 34 potential metabolic markers for arsenate toxicity.

Metabolite Name	KEGG	Kovats RI	Fiehn RI	VIP	Fold Change	p value	Status
Cholic acid	C00695	3418.5	1110668	1.79	1.51	0.0021	probable
Unknown (peak 311)		3436.6	1113712	1.69	1.41	0.0044	
Ursodeoxycholic acid	C07880	3301.8	1094022	1.64	1.31	0.0083	probable
Chenodeoxycholic acid	C02528	3344.6	1099922	1.64	1.33	0.0087	probable
Unknown (peak 152)		1710.5	583073	1.62	1.90	0.0037	
Unknown (peak 8)		1023.4	220331	1.61	1.66	0.0118	
Unknown (peak 314)		3523.3	1128266	1.59	1.32	0.0150	
Mannose	C00159	1953.8	686570	1.50	1.31	0.0113	probable
2-Ketovaline	C00141	1153.8	291985	1.47	1.75	0.0151	probable
Unknown (peak 189)		1943.8	682483	1.46	2.14	0.0252	
Unknown (peak 67)		1253.3	349182	1.39	1.86	0.0241	
Unknown (peak 59)		1219.7	329155	1.39	1.43	0.0266	
Gluconic acid	C00257	1946.4	683531	1.38	1.49	0.0246	probable
Unknown (peak 234)		2201.3	809327	1.36	1.45	0.0280	
Succinic acid	C00042	1305.7	380427	1.28	1.67	0.0429	confirmed
Unknown (peak 63)		1238.9	340611	1.28	2.08	0.0434	
Unknown (peak 140)		1619.3	541619	1.26	1.76	0.0482	
Glycylglycine	C02037	1814	629175	1.92	0.57	0.0002	probable
2-Oxovaleric acid	C06255	1111.3	268640	1.69	0.68	0.0029	probable
Myo-Inositol 1-phosphate	C01177	2487.3	885736	1.63	0.85	0.0218	probable
Unknown (peak 15)		1053.8	237000	1.62	0.60	0.0044	
Hypotaurine	C00519	1602.4	533953	1.60	0.63	0.0130	probable
3-hydroxybutanoic acid	C01089	1158.5	294592	1.60	0.59	0.0117	confirmed
Unknown (peak 4)		1002.7	208915	1.57	0.40	0.0084	
Palmitoleic acid	C08362	2016.7	711860	1.53	0.44	0.0193	confirmed
Glycine	C00037	1113.1	269628	1.52	0.69	0.0198	confirmed
Unknown (peak 101)		1431.3	447764	1.51	0.40	0.0213	
Ethanolamine	C00189	1243.3	343218	1.48	0.81	0.0447	confirmed
2-Aminobutanoate	C02356	1175.1	303717	1.47	0.56	0.0276	probable
Linoleic acid	C01595	2199.6	808238	1.46	0.62	0.0198	confirmed
Alpha-linolenic acid	C06427	2203.8	810927	1.46	0.44	0.0352	probable
Oleic acid	C00712	2208.5	813872	1.42	0.58	0.0280	probable
Unknown (peak 117)		1517.3	491580	1.37	0.72	0.0418	
Threonine	C00188	1294.5	373791	1.37	0.33	0.0447	confirmed

Potential markers were withVIP>1 in PLS-DA model and *p*<0.05 in Welch's t test.

### Ingenuity Pathway Analysis revealed liver damages at molecular level

**Fig 2:**
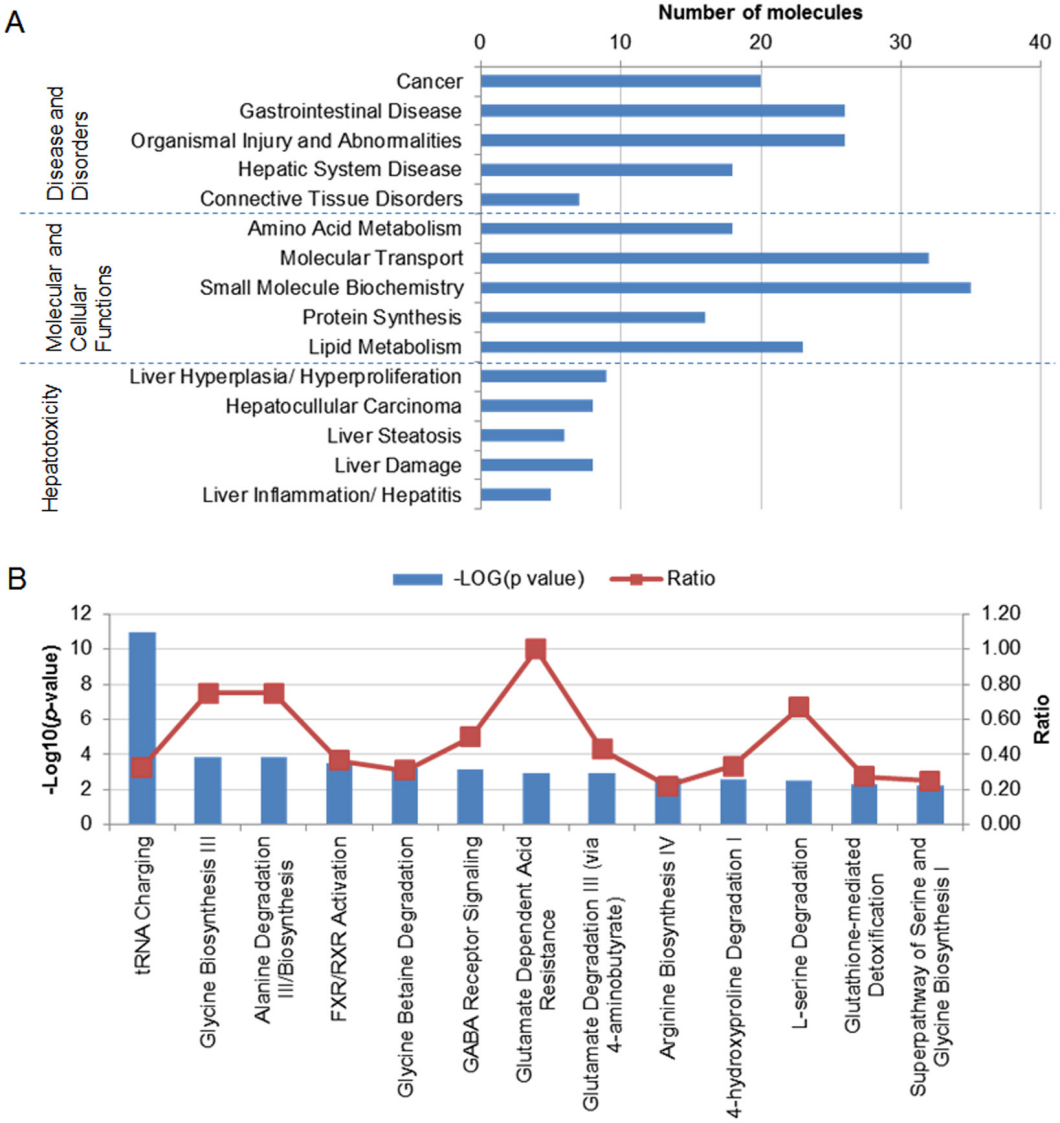
Effects of the metabolic alteration on Disease and Bio Functions and Tox Functions predicted by IPA (*p*<0.05). (A) The top five entries in three aspects of biological impact: Disease and Disorders, Molecular and Cellular Functions, and Hepatotoxicity. (B) Top altered canonical pathways predicted by IPA (p<0.005). The p values were calculated using Fischer's exact test determining the probability that the association between the genes in the data set and the canonical pathway was due to chance alone. The ratios were calculated based on the number of genes from the data set that mapped to the pathway and the total number of genes in the pathway.

To infer the possible biological impact associated with the metabolic changes, the KEGG IDs and log_2_ FC of 57 identified metabolites (VIP>1) were submitted to IPA for Metabolomic analysis. IPA predicted effects on Disease and Bio Functions and Tox Functions (Fig 2A, S2 Table). In the aspect of Disease and Disorders, top categories were Cancer and diseases in gastrointestinal and hepatic systems, suggesting damages to the liver. Nervous System Development and Function was also significant (*p* = 7.78E-08), consistent with the neurotoxic effects reported in zebrafish embryo/larvae [17]. In Molecular and Cellular Functions, Amino Acid Metabolism was altered with the top significance, followed by molecular transport (of small carbohydrates and lipids). Interestingly, a few annotation terms of Lipid Metabolism were also affected, including concentration of fatty acid, sterol, and cholesterol; and conversion of lipids. Arsenic-induced changes in lipid metabolism genes in zebrafish have been reported before [23]. Taken together, the output suggested that arsenic could damage the liver and affect the cellular transport and metabolism of amino acids and lipids. In terms of Hepatotoxicity predicted under Top Tox Functions, Liver Hyperplasia and Hepatocellular Carcinoma were the top predicted toxico-pathological outcomes, followed by Liver Seatosis and Liver Damage.

IPA also predicted 13 significantly altered canonical pathways (*p*<0.005, Fig 2B). The reduction of multiple amino acids led to significant predicted changes in pathways such as tRNA Charging and metabolism of specific amino acids (e.g. glycine biosynthesis/betaine degradation, alanine degradation/biosynthesis, and glutamate degradation via 4-aminobutyrate). Glutathione-mediated detoxification was also altered. An interesting pathway to note is FXR/RXR activation, which plays a role in modulation of bile acid homoeostasis, lipid metabolism and glucose [35].

### Histological examination showed that As weakened cellular organization, changed content of glycogen and lipid, and induced apoptosis

**Fig 3:**
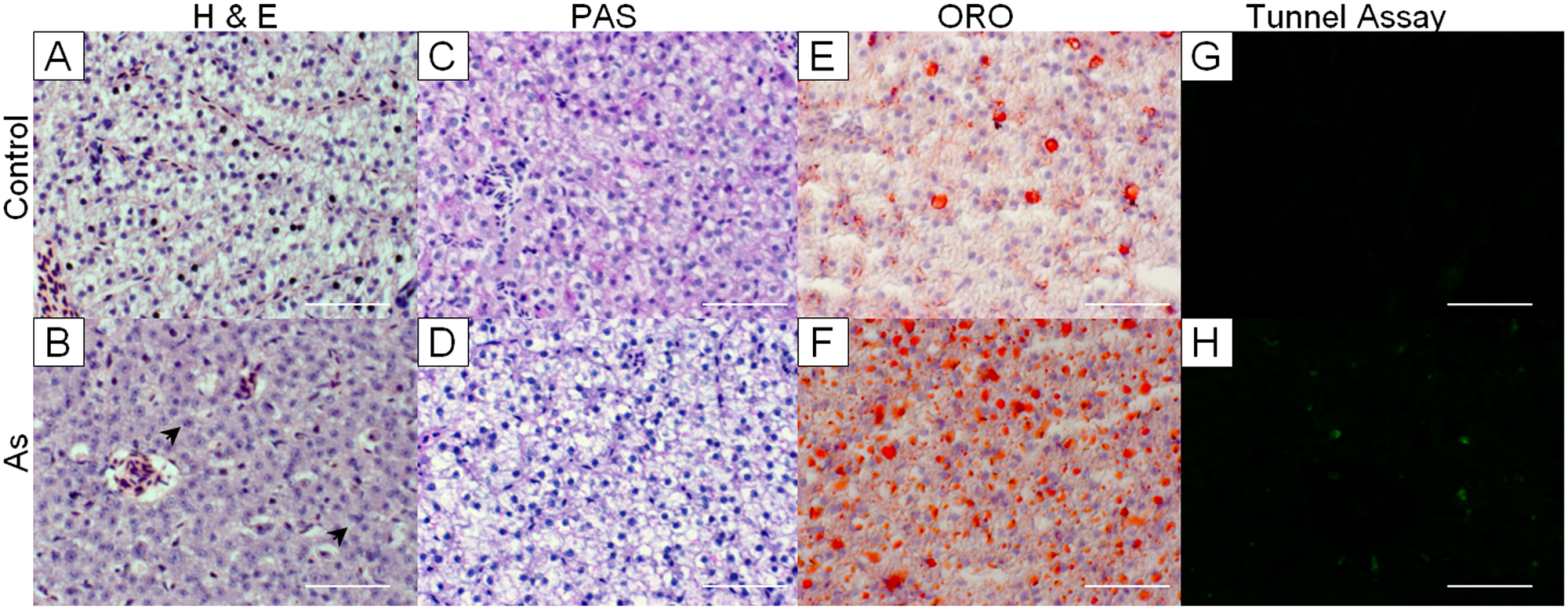
Histological examinations of liver sections. (A-B) H&E staining revealed changes in cellular organization, cytoplasmic volume, and nuclei morphology in treated group (lower panel) as compared to control (upper panel). (C-D) ORO staining showed marked accumulation of lipid dropolets in As group. (E-F) Glycogen depletion in the As group was evident with PAS staining. (G-H) Increased apoptotic cells (stained with green fluorescence) were observed in the As group. Magnification: 400x. Arrow: binucleated cells. Scale bars: 50μm.

Histological analyses were performed to validate liver damages predicted in IPA. In fact, at the end of arsenate exposure, livers of As-treated fish appeared to be softer and more watery than those of control fish, indicating compromised integrity of the tissue. In H&E stained sections, control hepatocytes were homogenous in size and highly organized into a two-cell plates (Fig 3A). In contrast, hepatocytes in treated group were less organized and more heterogeneous in size. Nuclei were generally larger and slightly more variable in size than those in controls and binucleated cells were occasionally observed. These changes were consistent with previous observations [24]. Empty spots of various sizes were observed in As-treated samples (Fig 3B), which might be a result of cell death and/or accumulation of fats as both of them showed significant increases (see Fig 3E–3H). Eosin stained area increased in the As group, suggesting reduction of glycogen contents. This was confirmed by PAS staining, where control livers were all stained reddish (Fig 3C) but at least 60% (6/9) of treated liver were less stained (Fig 3D). Depletion of liver glycogen after arsenate exposure has been described before and it was thought to be a result of glycogen mobilization induced by toxicant stress [24]. An additional cause may be the reduction of pentavalent arsenic to trivalent form in liver [36] by glycogen phosphorylase, a reaction that is coupled to glycogenolysis [37]. The product of glycogenolysis, Glucose-1-phosphate (G1P), can be converted into Glucose-6-phosphate (G6P) for glycolysis.

ORO staining was performed to visualize lipid content. Orange-red fat droplets were markedly increased in As group (Fig 3E and 3F). Interestingly, IPA predicted a negative z score on liver steatosis (S2 Table) based on the increase of fructose and reduction of oleic acid, glutamic acid, methionine, phenylalanine, and tryptophan. This seemed to be contradictory to the ORO staining results. While reduction of the amino acids and a fatty acid might be related to decreased lipogenesis, increase of fructose can increase glucose influx and contribute to *de novo* lipogeneis and insulin resistance [38], which could contribute to accumulation of hepatic fats. Liver stores excess lipids as neutral lipid esters in droplets to prevent lipotoxicity [39]. ORO stains cholesteryl esters and neutral lipids, including triglycerides (TGs) and diglycerides (DGs), on frozen tissue sections [40]. It is possible that upon arsenic treatment, fat accumulates in the liver as lipid esters in droplets while free fatty acids reduce or remain unchanged. In fact, two saturated fatty acids (octadecanoic acid and tetradecanoic acid) and one trans unsaturated fatty acid (elaidic acid) detected were not significantly altered (S3 Table). These differential effects of sodium arsenate on fatty acids are partially consistent with a recent study in male rats [41]. After 10 months of exposure to arsenic, cis unsaturated fatty acids significantly decreased, while saturated fatty acids and trans unsaturated fatty acid increased in treated group. Differential effects of saturated and unsaturated fatty acids on fatty liver progression have been reported before but underlying mechanisms remain unclear [42]. As excretion of bile acids is the primary way of cholesterol elimination [43], the accumulation of bile acids observed indicated compromised removal of cholesterols, which could in turn contribute to lipid accumulation.

Tunnel assay revealed notable increase of apoptotic cells in the treated livers (Fig 3G and 3H), confirming the indication of tissue damages by IPA. Arsenic-induced apoptosis has long been described and considered to be a result of a sequential event: early decrease in cellular mitochondria potential, increase of reactive oxygen species (ROS), and finally activation of caspase-3 dependent apoptosis pathway resulting DNA fragmentation [44–47]. These histopathological alterations confirmed that the current exposure is sufficient to cause some cellular damages in liver and apoptosis of a small percentage of hepatocytes. However, the liver impairments were still mild, as there were no severe features observed, such as inflammation, massive cell death, marked ballooning, cirrhosis and polymorphism of cell size and nuclei size (tumour features) [48–50]. Beside, accumulation of lipid droplets is reversible before fibrosis and cirrhosis have developed [50]. In fact, fibrosis was not observed in picrosirius red staining of the liver sections (data not shown).

### Conventional liver function tests did not indicate liver damages

**Fig 4:**
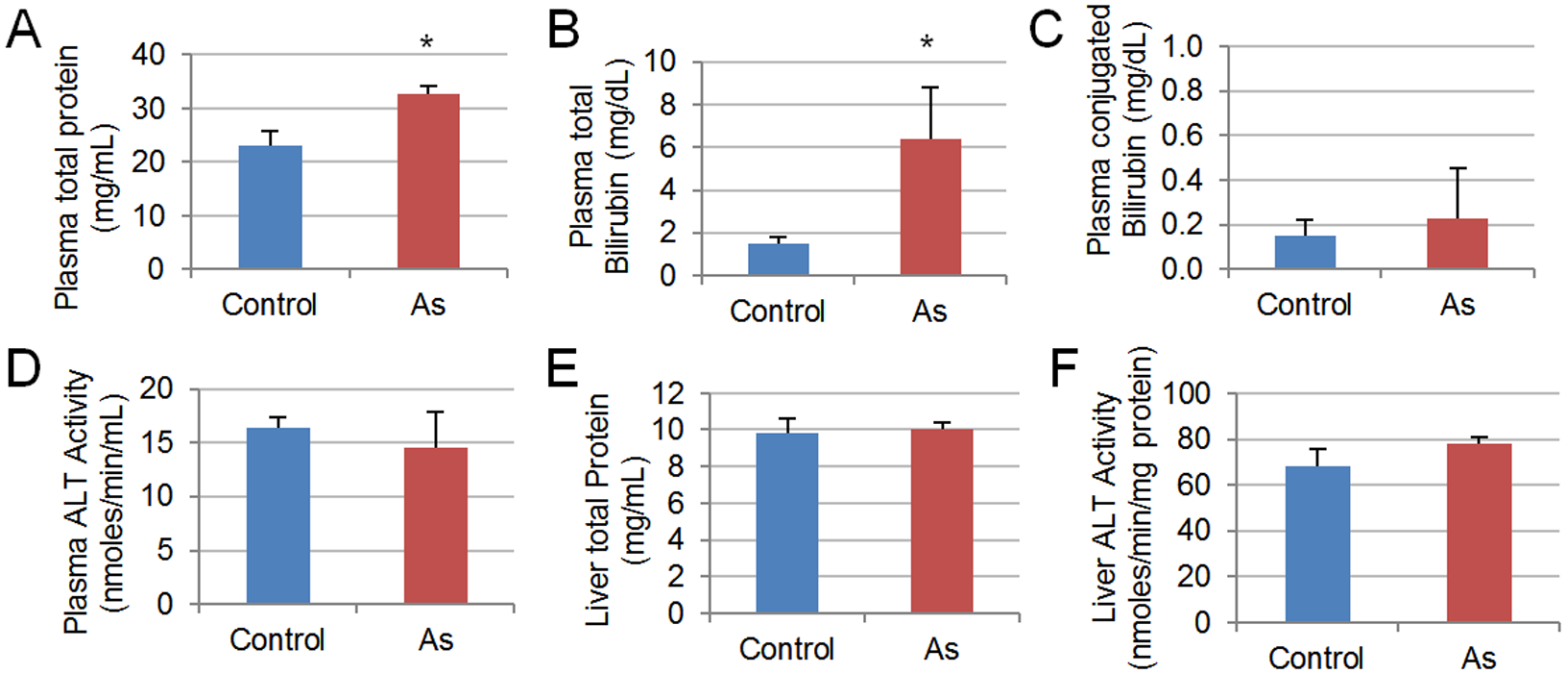
Liver function tests by Bilirubin assay (A-C) and ALT assay (D-F). Total protein content (A) and total bilirubin (B) were significantly higher in plasma of As-treated fish as compared to control, with *p* = 0.008 and *p* = 0.037 respectively. Direct bilirubin (C) and plasma ALT activity (D) remained normal (*p>0*.*3*). No significant change was found in total protein concentration (E) or ALT activity (F) of liver (*p*>0.1). Five biological replicates were used for each assay and statistical test used was one-tailed student *t*-test.

To further assess the liver damages, we performed two conventional liver function tests, plasma bilirubin assay and ALT activity assay. Plasma bilirubin level can be altered by conditions such as hepatitis, biliary obstruction and cirrhosis, while plasma ALT level elevates if integrity of hepatocytes is compromised [51]. Five biological replicates, each a pooled sample from 3–6 fish, were used in all assays (Fig 4).

Unconjugated bilirubin is a product of heme catabolism, which can be transported to the liver and conjugated to glucuronides by UDP-glucuronosyltransferases (UGTs) to form conjugated bilirubin. Conjugated bilirubin is secreted into bile and excreted or recycled to plasma, accounting for a minute percentage of total plasma bilirubin [52]. Results showed that total plasma protein was elevated by about 35% (*p*<0.01) in the As group (Fig 4A). Total bilirubin in the As group was 6.42±5.43 mg/dL, significantly higher (*p*<0.05) than the value of 1.73±0.94 mg/dL in controls (Fig 4B). Conjugated bilirubin was similarly low or absent in both As (0.23±0.51 mg/dL) and control (0.13±0.15 mg/dL) samples (Fig 4C), indicating absence of bile duct obstruction, hepatitis or cirrhosis [51]. An increase in unconjugated bilirubin is likely a result of hemolysis and/or ineffective bilirubin conjugation. Bilirubin conjugation in zebrafish has not been well studied. A recent *in vitro* study has shown that only *ugt1* family could conjugate bilirubin, among which *ugt1b7* has the highest catalytic activity and it is detected in the liver [53]. However, in a liver transcriptomic profile we obtained under the same As exposure conditions, none of the *ugt1* genes is deregulated [3]. Furthermore, there has been reports of hemolytic anaemia in individuals with acute [54] or chronic [55] exposure. Thus, it is more probable that the elevation of unconjugated bilirubin was due to increased hemolysis in blood.

ALT is highly enriched in the liver and liver injuries lead to increase in its serum concentration [51]. Unexpectedly, plasma ALT activity was not significantly altered (*p*>0.3, Fig 4D). Total protein and ALT activity were also quantified in liver samples and no significant change was observed (Fig 4E and 4F). Taken together, the analysis suggested there was probably no severe damage of liver. It should be pointed out that, plasma used in these function assays were obtained from fish exposure to 15 ppm and greater changes might be observable at 20 ppm. However, exposure to arsenic does not necessarily led to significant changes in plasma live function assays. In a study on a population of west Bengal of India exposure to different levels of arsenic, serum bilirubin was elevated in total exposed individuals but no significance was detected in dose-response tests [56]. ALT activity in plasma were frequently elevated after arsenic exposure in human [56, 57] or animal models [58, 59]; however, such changes are usually significant only under very high concentrations [57, 60] or prolonged exposure of weeks to months[61–63]. Thus, the arsenate exposure in this study was probably not sufficient to induce severe liver damages. This is further supported by the absence of histopathological evidence for severe liver damages as described earlier. In addition, it has been recently recognized that, ALT level may remain normal in patients with non-alcoholic fatty liver disease, viral hepatitis and some metabolic disorders [64].

### Arsenate altered metabolic networks of bile acid regulation, glycolysis, and fatty acid metabolism

The experimental evidence, knowledge-based data mining of biological pathways and associated biological interpretations together indicated that, the toxicity exerted by arsenate involved bile acid homeostasis, amino acid metabolism, glycogen metabolism and lipid metabolism. We further examined a network focusing glycogenolysis and glycolysis pathway, which is central to energy metabolism and also related to amino acid and lipid metabolism, with reference to relevant mRNA changes from a transcriptomic profile of arsenate exposure we previously obtained using the same exposure conditions [3]. The network structure was modified from IPA Target Explorer (https://targetexplorer.ingenuity.com/index.htm).

**Fig 5:**
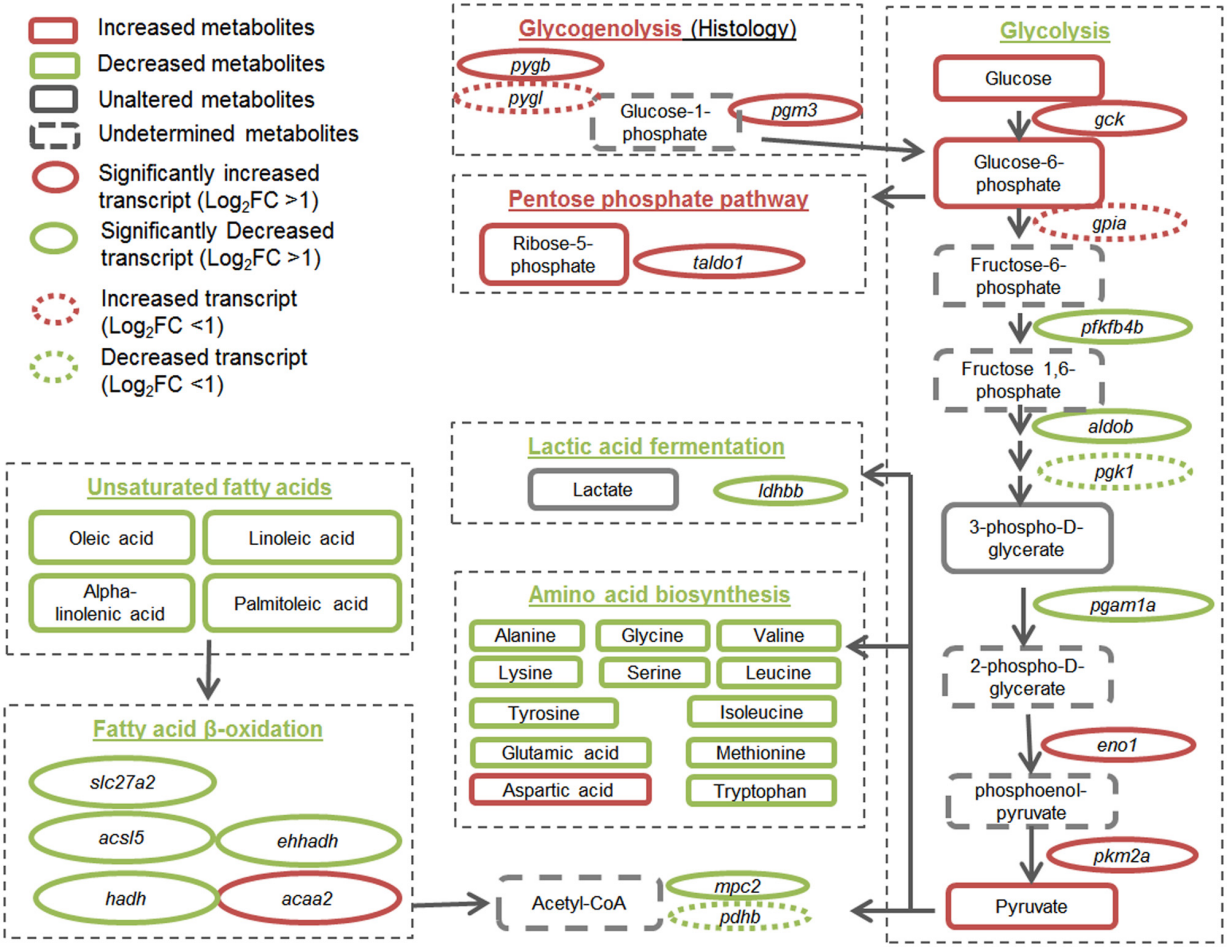
Network of glycolysis, glycogen metabolism and fatty acid metabolism constructed based on metabolic changes found in this study and mRNA changes reported in our previous transcriptomic study [3]. Gene names: *acaa2*, acetyl-CoA acyltransferase 2; *acsl5*, acyl-CoA synthetase long-chain family member 5; *aldob*, aldolase b, fructose-bisphosphate; *ehhadh*, enoyl-Coenzyme A, hydratase/3-hydroxyacyl Coenzyme A dehydrogenase; *eno1*, enolase 1, (alpha); *gck*, glucokinase (hexokinase 4); *gpia*, glucose phosphate isomerase a; *hadh*, hydroxyacyl-Coenzyme A dehydrogenase; *ldhbb*, lactate dehydrogenase Bb; *mpc2*, mitochondrial pyruvate carrier 2; *pfkfb4b (pfkfb4l)*, 6-phosphofructo-2-kinase/fructose-2,6-biphosphatase 4b; *pgam1a*, phosphoglycerate mutase 1a; *pgm3*, phosphoglucomutase 3; *pgk1*, phosphoglycerate kinase 1; *pkm2a*, pyruvate kinase, muscle, a; *pygb*, phosphorylase, glycogen; brain; *Pygl*, phosphorylase, glycogen, liver; *slc27a2*, solute carrier family 27 (fatty acid transporter), member 2; *taldo1*, transaldolase 1.

The observation of arsenic-induced glycogenolysis and glycolysis intermediates with concurrent increase in pyruvate has been reported before [65]. Besides, mechanism of arsenic toxicity has been postulated to include inhibition of energy-linked functions in mitochondria, direct inhibition of pyruvate dehydrogenase, and disruption of enzymatic reaction involving glyceraldehyde 3-phosphate dehydrogenase (GAPDH) [36]. As shown in Fig 5, the first and last few steps of glycolysis seemed to be promoted while intermediate steps were down-regulated. In addition to increased glucose, glycogenolysis (evident in the PAS staining) and increased conversion of G1P to G6P together provided more input to glycolysis, which was supported by the up-regulation of transcripts for key enzymes in the process (*pygb*, *pgm3 and pybl*). However, G6P seemed to be channeled to pentose phosphate pathway (PPP) and resulted in increase in ribose-5-phosphate (R5P), a substrate for synthesis of ribonucleotides. The PPP is also a major source of NADPH, which is a scavenger of ROS and is required for fatty acid synthesis [66]. The transcriptomic profile showed no increase in fatty acid or ribonucleotide synthesis pathway, so this channeling to PPP may be favored to counter the oxidative stress induced by arsenic. Interestingly, pyruvate was built up and none of the downstream utilization seemed to be activated: transcript of the key enzyme *ldhbb* for lactic acid fermentation was down-regulated; amino acids biosynthesis seemed to be impaired; and transport of pyruvate to mitochondria and to acetyl-CoA were both inhibited (down-regulation of *mpc2* and *pdhb)*. The insufficient utilization of pyruvate might be a protective response of the cell to avoid generation of more ROS in the catabolism of acetyl-CoA. Not only the conversion of pyruvate to acetyl-CoA was reduced, oxidation of fatty acid at mitochondria was also down-regulated at the transcript level, suggesting a reduction in another source of acetyl-CoA to fuel the TCA cycle for energy production. It is also noted that genes coding for enzymes that hydrolyze TGs and cholesteryl esters (*cel*.*1* and *cel1*) were also down-regulated, which may partially account for the reduction in free unsaturated fatty acids and increased lipid droplets.

Interestingly, the changes in glycolysis pathway partially resembled Warburg effect, a metabolic feature commonly observed in many cancer cells, where there is enhanced glycolysis and lactate fermentation even in presence of sufficient oxygen [67]. It has been reported that Warburg effect provides cancer cells with growth advantages (increased building blocks for biosynthesis and reduced oxidative stress) [68]. Rate limiting enzymes in glycolysis and lactate fermentation pathway, including glucokinases, pyruvate kinase and lactate dehydrogenase, are up-regulated in Warburg effect; in addition, PPP and TCA cycle are also enhanced [69, 70]. In arsenate treated liver, we observed increased transcripts of glucokinase (*gck*) and pyruvate kinase (*pkm2a*), increased G6P and pyruvate, and increased activity of PPP. In particular, *pkm2a* is coding for the M2 isoform of pyruvate kinases, an isoform whose expression is not normally found in the liver but is elevated in cancer cells [71–73]. The main difference from Warburg effect was the lack of enhanced lactate fermentation, based on the decrease of lactate as well as down-regulation of *ldhbb*. Arsenic-induced Warburg effect has been reported in only one *in vitro* study [74], where sodium arsenite increased glycolysis and lactate fermentation in human bronchial epithelial cells. Interestingly, while transcripts for enzymes in all glycolytic steps increased at 2 weeks and 4 weeks of exposure, transcripts of some enzymes at the intermediate steps (eg. ENO3 and PGK2) decreased after 1 week of exposure while transcripts for GCK. This is consistent with our observations described. We postulate that, with prolonged exposure, arsenate might also induce Warburg effect as a secondary consequence of direct toxic effects, which could in turn contribute to carcinogenesis.

Taken together, we hypothesize that cellular perturbations in liver by acute exposure to sodium arsenate may involve the following aspects: (1) glycogen is rapidly depleted, with at least some of the end products entering glycolysis; (2) G6P is mainly channeled to PPP, presumably for production of NADPH to counter the oxidative stress induced by arsenate; (3) pyruvate transport to mitochondria and further utilization are impaired and energy production is compromised, which could be a result of enzyme inhibition or a protective response to reduce generation of ROS; (4) various free amino acids are decreased; (5) bile acids accumulate in liver (risk of cholestasis); (6) cellular fatty acid composition is affected and lipid droplets dramatically increased, which could be a result of TG built up and compromised lipoprotein metabolism. The alterations in glycolysis pathway partially resemble Warburg effect, which could be a contributing factor of arsenate-induced carcinogenesis. In addition, this study indicated that metabolic changes could be sensitive markers to reflect mild liver impairments (e.g. induced by arsenic exposure), which may not be detectable by conventional liver function tests such as Bilirubin assay and ALT assay.

## Supporting Information

S1 Fig Hierarchical clustering of samples using peak areas of subset of seven metabolites. Peak areas were quantile-normalized and standardized to respective mean value of each metabolite among all samples. Hierarchical clustering was performed using Pearson correlation method without mean centering.
(TIF)

S2 Fig Standard curve of calibrator for bilirubin assay in recommended assay setup and the scaled-down setup used in this study. Limited by the minute amount of plasma collectable from zebrafish, assay was scaled down (2 μL plasma in 125 μL assay mixture) from the manufacturer’s protocol (50 μL in 250 μL assay mixture). The linearity was conserved across the two assay setups and thus the use of the modified assay setup was valid.
(TIF)

S1 Table Complete list of 57 altered metabolites (contributing to group separation in PLS-DA model) identified by GC/MS. (DOCX)

S2 Table IPA predicted effects of the metabolic alteration on Disease and Biofunctions and Tox Functions. (*p*<0.01 and #molecular> = 3) (XLSX)

S3 Table List of unaltered metabolites (not contributing to group separation in PLS-DA modeling) identified by GC/MS. (DOCX)
