# Supplementary figures and images for: Metabolomic Characterizations of Liver Injury Caused by Acute Arsenic Toxicity in Zebrafish
Source: PLoS One. 2016 Mar 11;11(3):e0151225. doi: 10.1371/journal.pone.0151225 (PMC4788152; doi:10.1371/journal.pone.0151225)

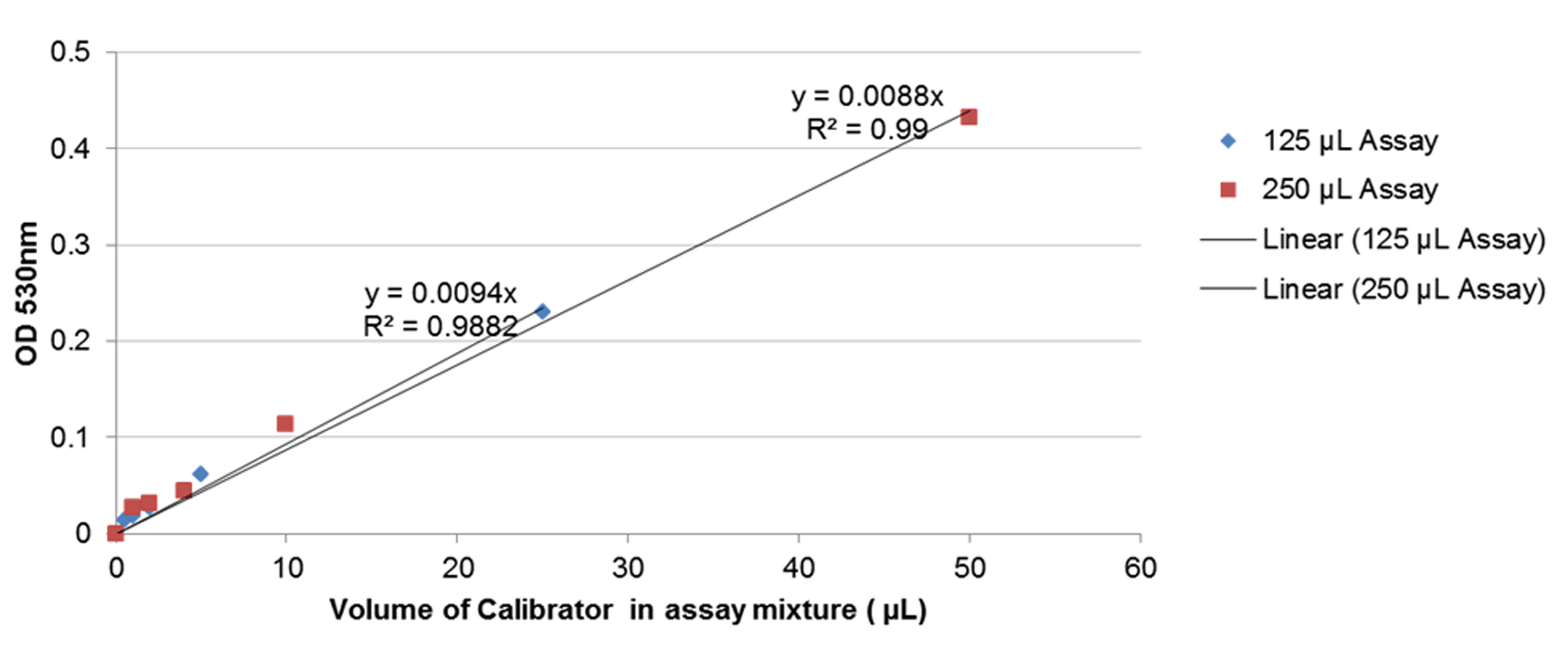

Supplement: S1 Fig — Peak areas were quantile-normalized and standardized to respective mean value of each metabolite among all samples. Hierarchical clustering was performed using Pearson correlation method without mean centering. (TIF) [file pone.0151225.s001.tif]

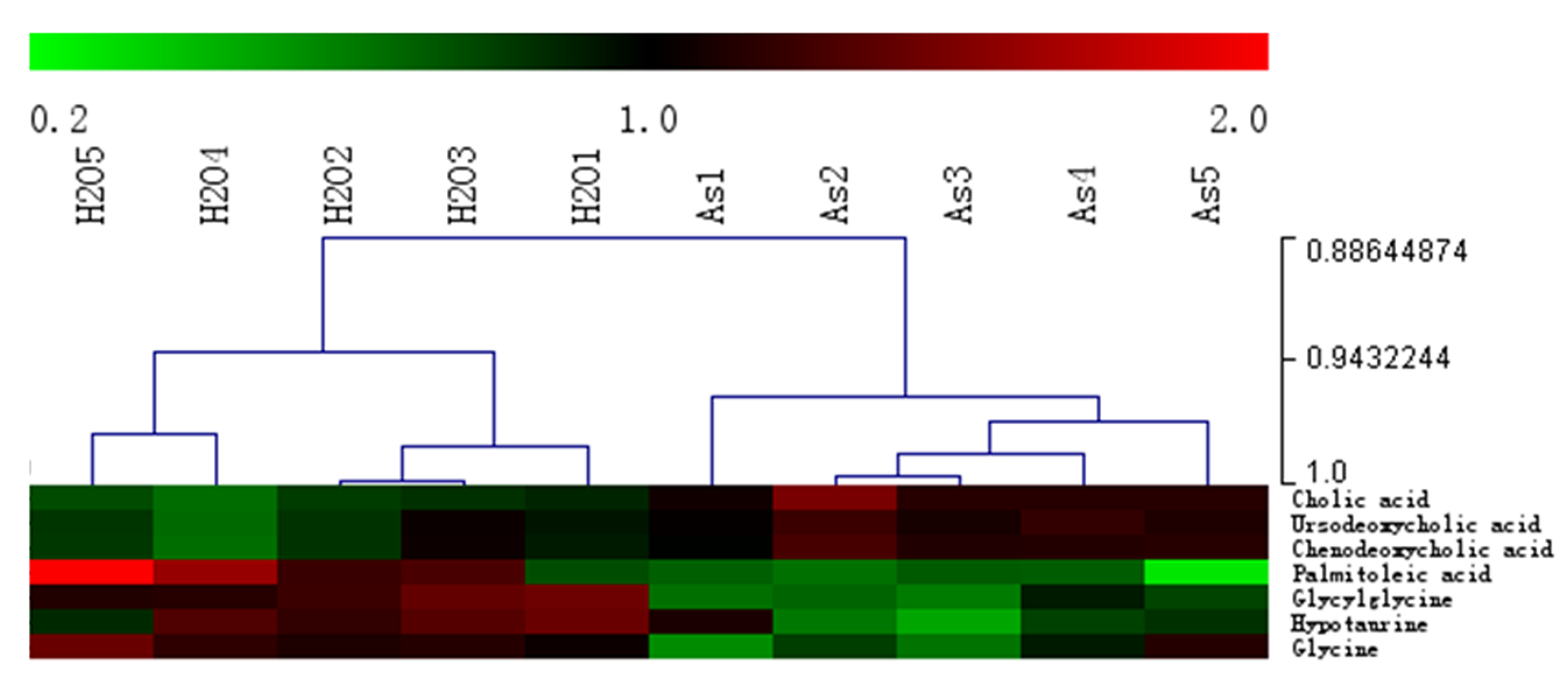

Supplement: S2 Fig — Limited by the minute amount of plasma collectable from zebrafish, assay was scaled down (2 μL plasma in 125 μL assay mixture) from the manufacturer’s protocol (50 μL in 250 μL assay mixture). The linearity was conserved across the two assay setups and thus the use of the modified assay setup was valid. (TIF) [file pone.0151225.s002.tif]
