# Supplementary material for: Metabolomic Characterizations of Liver Injury Caused by Acute Arsenic Toxicity in Zebrafish
Source: PLoS One. 2016 Mar 11;11(3):e0151225. doi: 10.1371/journal.pone.0151225 (PMC4788152; doi:10.1371/journal.pone.0151225)
Supplement: S1 Table — (DOCX) [file pone.0151225.s003.docx]

**S1 Table.** **Complete list of 57 altered metabolites (contributing to group separation in PLS-DA model) identified by GC/MS.**

| **Metabolite Name** | **KEGG** | **Formula** | **Kovats RI** | **Fiehn RI** | **Derivative Status** | **VIP** | **Fold change** | **p value** | **Status** |
| --- | --- | --- | --- | --- | --- | --- | --- | --- | --- |
| Glycylglycine | C02037 | C4H8N2O3 | 1814 | 629175 | 3TMS | 1.92 | 0.57 | 0.0002 | probable |
| Cholic acid | C00695 | C24H40O5 | 3418.5 | 1110668 | N.A. | 1.79 | 1.51 | 0.0021 | probable |
| 2-Oxovaleric acid | C06255 | C5H8O3 | 1111.3 | 268640 | 1MEOX1TMS | 1.69 | 0.68 | 0.0029 | probable |
| Ursodeoxycholic acid | C07880 | C24H40O4 | 3301.8 | 1094022 | 3TMS | 1.64 | 1.31 | 0.0083 | probable |
| Chenodeoxycholic acid | C02528 | C24H40O4 | 3344.6 | 1099922 | 3TMS | 1.64 | 1.33 | 0.0087 | probable |
| Myo-Inositol 1-phosphate | C01177 | C6H13O9P | 2487.3 | 885736 | 7TMS | 1.63 | 0.85 | 0.0218 | probable |
| Hypotaurine | C00519 | C2H7NO2S | 1602.4 | 533953 | 3TMS | 1.60 | 0.63 | 0.0130 | probable |
| 3-hydroxybutanoic acid | C01089 | C4H8O3 | 1158.5 | 294592 | 2TMS | 1.60 | 0.59 | 0.0117 | confirmed |
| Palmitoleic acid | C08362 | C16H30O2 | 2016.7 | 711860 | 1TMS | 1.53 | 0.44 | 0.0193 | confirmed |
| Glycine | C00037 | C2H5NO2 | 1113.1 | 269628 | 2TMS | 1.52 | 0.69 | 0.0198 | confirmed |
| Mannose | C00159 | C6H12O6 | 1953.8 | 686570 | 1MEOX5TMS | 1.50 | 1.31 | 0.0113 | probable |
| Ethanolamine | C00189 | C2H7NO | 1243.3 | 343218 | 3TMS | 1.48 | 0.81 | 0.0447 | confirmed |
| 2-Aminobutanoate | C02356 | C4H9NO2 | 1175.1 | 303717 | 2TMS | 1.47 | 0.56 | 0.0276 | probable |
| 2-Ketovaline | C00141 | C5H8O3 | 1153.8 | 291985 | 1MEOX1TMS | 1.47 | 1.75 | 0.0151 | probable |
| Linoleic acid | C01595 | C18H32O2 | 2199.6 | 808238 | 1TMS | 1.46 | 0.62 | 0.0198 | confirmed |
| Alpha-linolenic acid | C06427 | C18H30O2 | 2203.8 | 810927 | 1TMS | 1.46 | 0.44 | 0.0352 | probable |
| Oleic acid | C00712 | C18H34O2 | 2208.5 | 813872 | 1TMS | 1.42 | 0.58 | 0.0280 | probable |
| Gluconic acid | C00257 | C6H12O7 | 1946.4 | 683531 | 1MEOX5TMS | 1.38 | 1.49 | 0.0246 | probable |
| Threonine | C00188 | C4H9NO3 | 1294.5 | 373791 | 2TMS | 1.37 | 0.33 | 0.0447 | confirmed |
| D-Ribose 5-phosphate | C00117 | C5H11O8P | 2139.4 | 766754 | 5TMS | 1.36 | 1.38 | 0.0577 | probable |
| Methylmalonic acid | C02170 | C4H6O4 | 1190.3 | 312052 | 2TMS | 1.33 | 0.56 | 0.0632 | probable |
| Cadaverine | C01672 | C5H14N2 | 1861.2 | 648529 | 4TMS | 1.32 | 0.19 | 0.0688 | probable |
| Hydroxyproline | C01157 | C5H9NO3 | 1523 | 494464 | 3TMS | 1.30 | 0.40 | 0.0591 | confirmed |
| Valine | C00183 | C5H11NO2 | 1071.8 | 246915 | 1TMS | 1.29 | 0.34 | 0.0549 | confirmed |
| Erythrono-1,4-lactone | N.A. | C4H6O4 | 1413.8 | 438836 | 2TMS | 1.29 | 0.21 | 0.0783 | probable |
| Succinic acid | C00042 | C4H6O4 | 1305.7 | 380427 | 2TMS | 1.28 | 1.67 | 0.0429 | confirmed |
| Tyrosine | C00082 | C9H11NO3 | 1872.8 | 653306 | 2TMS | 1.26 | 0.25 | 0.0867 | confirmed |
| Lithocholic acid | C03990 | C24H40O3 | 3246.4 | 1086388 | 2TMS | 1.25 | 1.20 | 0.0510 | probable |
| D-Fructose | C00095 | C6H12O6 | 1795.9 | 621572 | 1MEOX5TMS | 1.23 | 1.26 | 0.0558 | probable |
| 4-aminobutanoic acid | C00334 | C4H9NO2 | 1565.5 | 515633 | 3TMS | 1.22 | 1.92 | 0.0737 | probable |
| Niacinamide | C00153 | C6H6N2O | 1444.6 | 454568 | No | 1.21 | 0.83 | 0.0702 | probable |
| Serine | C00065 | C3H7NO3 | 1252.8 | 348906 | 2TMS | 1.20 | 0.08 | 0.1072 | confirmed |
| Inosine | C00294 | C10H12N4O5 | 2583.7 | 908916 | 4TMS | 1.19 | 0.82 | 0.1115 | confirmed |
| L-Glutamic acid | C00025 | C5H9NO4 | 1521.4 | 493655 | 2TMS | 1.18 | 0.34 | 0.0808 | Confirmed |
| Citrulline | C00327 | C6H13N3O3 | 1729.8 | 591791 | N.A. | 1.17 | 0.33 | 0.1229 | probable |
| Tryptophan | C00078 | C11H12N2O2 | 2179.3 | 794218 | 2TMS | 1.17 | 0.33 | 0.1221 | confirmed |
| D-Mannonate | C00514 | C6H12O7 | 1913.1 | 669838 | 4TMS | 1.16 | 1.44 | 0.0872 | confirmed |
| Aminomalonic acid | C00872 | C3H5NO4 | 1475.8 | 470516 | 3TMS | 1.16 | 2.95 | 0.0987 | probable |
| Aspartic acid | C00049 | C4H7NO4 | 1482.2 | 473792 | 3TMS | 1.15 | 1.29 | 0.0889 | confirmed |
| 2-Hydroxypyridine | C02502 | C5H5NO | 1021.3 | 219146 | 1TMS | 1.15 | 1.16 | 0.1335 | probable |
| Methionine | C00073 | C5H11NO2S | 1518 | 491967 | 2TMS | 1.15 | 0.30 | 0.1252 | confirmed |
| Isoleucine | C00407 | C6H13NO2 | 1303.3 | 379005 | 2TMS | 1.14 | 0.34 | 0.1205 | confirmed |
| Alanine | C00041 | C3H7NO2 | 1099.8 | 262320 | 2TMS | 1.14 | 0.14 | 0.1322 | confirmed |
| Ornithine | C00077 | C5H12N2O2 | 1760.5 | 605601 | 3TMS | 1.13 | 0.29 | 0.1455 | confirmed |
| Oxalic acid | C00209 | C2H2O4 | 1131.1 | 279503 | 2TMS | 1.12 | 2.35 | 0.0969 | confirmed |
| Phenylalanine | C00079 | C9H11NO2 | 1523.8 | 494851 | 1TMS | 1.11 | 0.28 | 0.1448 | confirmed |
| 3-Hydroxy-2-methylbutanoic acid | N.A. | C5H10O3 | 1233.8 | 337530 | 2TMS | 1.10 | 1.32 | 0.1073 | probable |
| Lysine | C00047 | C6H14N2O2 | 1950.7 | 685312 | N.A. | 1.08 | 0.32 | 0.1631 | probable |
| Maltose | C00208 | C12H22O11 | 2791.6 | 969411 | 8TMS | 1.08 | 1.44 | 0.1452 | probable |
| Glucose-6-phosphate | C00092 | C6H13O9P | 2370.4 | 881732 | 6TMS | 1.07 | 1.29 | 0.1281 | probable |
| Leucine | C00123 | C6H13NO2 | 1280.7 | 365536 | 2TMS | 1.07 | 0.13 | 0.1697 | confirmed |
| Glucose | C00031 | C6H12O6 | 1958.9 | 688666 | 5TMS1MEOX | 1.05 | 1.43 | 0.1692 | probable |
| Ribitol | C00474 | C5H12O5 | 1756.1 | 603648 | 5TMS | 1.05 | 1.25 | 0.1409 | probable |
| Mannoic lactone | N.A. | C6H10O6 | 1988.9 | 700996 | 4TMS | 1.04 | 0.76 | 0.4694 | probable |
| Spermidine | C00315 | C7H19N3 | 2180.7 | 795178 | 4TMS | 1.02 | 0.62 | 0.1551 | probable |
| Pyruvic acid | C00022 | C3H4O3 | 1029.5 | 223688 | 1MEOX1TMS | 1.02 | 1.36 | 0.1305 | confirmed |
| Ribose | C00121 | C5H10O5 | 1632.6 | 547702 | 4TMS1MEOX | 1.01 | 1.34 | 0.2955 | probable |
