# Supplementary material for: Metabolomic Characterizations of Liver Injury Caused by Acute Arsenic Toxicity in Zebrafish
Source: PLoS One. 2016 Mar 11;11(3):e0151225. doi: 10.1371/journal.pone.0151225 (PMC4788152; doi:10.1371/journal.pone.0151225)
Supplement: S3 Table — (DOCX) [file pone.0151225.s005.docx]

**S3 Table**. **List of unaltered metabolites (not contributing to group separation in PLS-DA modeling) identified by GC/MS.**

| **Peak NO.** | **Metabolite Name** | **KEGG** | **Formula** | **Kovats RI** | **Fiehn RI** | **Derivative Status** | **VIP** | **Fold Change** | **p value** | **Status** |
| --- | --- | --- | --- | --- | --- | --- | --- | --- | --- | --- |
| 128 | Creatinine | C00791 | C4H7N3O | 1559.2 | 512503 | 3TMS | 0.93 | 0.79 | 0.2137 | Confirmed |
| 107 | 3-Aminoisobutanoic acid | C05145 | C4H9NO2 | 1473.2 | 469148 | 3TMS | 0.91 | 1.79 | 0.1972 | Confirmed |
| 225 | Heptadecanoic acid |  | C17H34O2 | 2140.8 | 767714 | 1TMS | 0.91 | 1.11 | 0.3593 | Confirmed |
| 172 | Tetradecanoic acid | C06424 | C14H28O2 | 1844.9 | 641833 | 1TMS | 0.85 | 0.84 | 0.2185 | Confirmed |
| 79 | Proline | C00148 | C5H9NO2 | 1299.1 | 376517 | 2TMS | 0.85 | 1.23 | 0.2507 | Confirmed |
| 167 | Phosphoethanolamine | C00346 | C2H8NO4P | 1796.8 | 621991 | 4TMS | 0.83 | 0.88 | 0.2630 | Confirmed |
| 89 | Fumaric acid | C00122 | C4H4O4 | 1343.9 | 401252 | 2TMS | 0.77 | 1.40 | 0.2918 | Confirmed |
| 163 | Putrescine | C00134 | C4H12N2 | 1769.7 | 609786 | 4TMS | 0.71 | 0.85 | 0.4340 | Confirmed |
| 83 | Glycine | C00037 | C2H5NO2 | 1316.3 | 386276 | 3TMS | 0.68 | 1.19 | 0.3590 | Confirmed |
| 42 | Benzyl alcohol | C00556 | C7H8O | 1138.5 | 283571 | 1TMS | 0.67 | 1.39 | 0.3721 | Confirmed |
| 88 | Glyceric acid | C00258 | C3H6O4 | 1342.5 | 400496 | 3TMS | 0.66 | 1.10 | 0.5627 | Confirmed |
| 206 | Xanthine | C00385 | C5H4N4O2 | 2000.2 | 705642 | 3TMS | 0.65 | 0.98 | 0.8806 | Confirmed |
| 113 | Malic acid | C00149 | C4H6O5 | 1500.1 | 482936 | 3TMS | 0.64 | 0.95 | 0.6701 | Confirmed |
| 115 | Pyroglutamic acid | C01879 | C5H7NO3 | 1507.3 | 486572 | 2TMS | 0.64 | 1.08 | 0.8067 | Confirmed |
| 124 | 4-Aminobutanoic acid | C00334 | C4H9NO2 | 1541.8 | 503817 | 3TMS | 0.63 | 1.25 | 0.4806 | Confirmed |
| 102 | Beta-Alanine | C00099 | C3H7NO2 | 1438.1 | 451256 | 3TMS | 0.52 | 0.89 | 0.5215 | Confirmed |
| 213 | Palmitic acid | C00249 | C16H32O2 | 2042 | 721361 | 1TMS | 0.48 | 0.88 | 0.6151 | Confirmed |
| 155 | Ribitol | C00474 | C5H12O5 | 1721 | 587816 | 5TMS | 0.46 | 0.92 | 0.6548 | Confirmed |
| 220 | Heptadecanoic acid |  | C17H34O2 | 2105.9 | 745394 | 1TMS | 0.44 | 1.05 | 0.8280 | Confirmed |
| 187 | Galactose | C00124 | C6H12O6 | 1942.7 | 682029 | 1MEOX 5TMS | 0.39 | 1.08 | 0.7161 | Confirmed |
| 17 | Lactic Acid | C00186 | C3H6O3 | 1056 | 238224 | 2TMS | 0.38 | 1.08 | 0.7150 | Confirmed |
| 159 | Glycerol 3-phosphate | C03189 | C3H9O6P | 1749.4 | 600614 | 4TMS | 0.27 | 1.03 | 0.7973 | Confirmed |
| 164 | Hypoxanthine | C00262 | C5H4N4O | 1779.9 | 614354 | 2TMS | 0.23 | 0.98 | 0.8010 | Confirmed |
| 58 | Urea | C00086 | CH4N2O | 1217.7 | 327970 | 2TMS | 0.18 | 0.96 | 0.8139 | Confirmed |
| 48 | Ketoleucine |  | C6H10O3 | 1163.1 | 297120 | 1TMS | 0.75 | 0.92 | 0.2894 | Probable |
| 214 | Dopamine | C03758 | C8H11NO2 | 2048.9 | 723946 | 4TMS | 0.74 | 0.93 | 0.4858 | Probable |
| 75 | Methylsuccinic acid |  | C5H8O4 | 1287.5 | 369565 | 2TMS | 0.70 | 1.01 | 0.9175 | Probable |
| 168 | 5-Aminolevulinic acid | C00430 | C5H9NO3 | 1800.7 | 623700 | 3TMS | 0.69 | 0.90 | 0.4748 | Probable |
| 286 | Maltose | C00208 | C12H22O11 | 2770.5 | 964250 | 8TMS | 0.63 | 1.24 | 0.3690 | Probable |
| 85 | Pyrimidine | C00396 | C4H4N2 | 1328.6 | 392972 | 2TMS | 0.58 | 1.07 | 0.4229 | Probable |
| 243 | Octadecanoic acid | C01530 | C18H36O2 | 2239.4 | 833462 | 1TMS | 0.52 | 0.91 | 0.7182 | Probable |
| 255 | Arachidonic acid | C00219 | C20H32O2 | 2358.4 | 881296 | 1TMS | 0.50 | 0.88 | 0.5176 | Probable |
| 268 | Serotonin | C00780 | C10H12N2O | 2504 | 886297 | 3TMS | 0.34 | 0.94 | 0.6676 | Probable |
| 279 | Glycerol 1-palmitate |  | C19H38O4 | 2598 | 913466 | 2TMS | 0.30 | 0.96 | 0.8906 | Probable |
| 254 | Arachidonic acid | C00219 | C20H32O2 | 2354.2 | 881145 | 1TMS | 0.29 | 0.99 | 0.9719 | Probable |
| 240 | Elaidic acid | C01712 | C18H34O2 | 2216 | 818610 | 1TMS | 0.28 | 1.00 | 0.9738 | Probable |
| 29 | 1-Octanol | C00756 | C8H18O | 1103.4 | 264295 | 1TMS | 0.18 | 1.01 | 0.9397 | Probable |
| *170 | Glycerate 3-phosphate | C00597 | C3H7O7P | 1823.6 | 633115 | N.A. | - | - | - | Probable |
| *301 | Cholesterol | C00187 | C27H46O | 3159.8 | 1069453 | 1TMS | - | - | - | Confirmed |

* Metabolites were not included in chemometric analysis due to large CV in QC samples.
